# Cereal Crops Are not Created Equal: Wheat Consumption Associated with Obesity Prevalence Globally and Regionally

**DOI:** 10.3934/publichealth.2016.2.313

**Published:** 2016-05-20

**Authors:** Wenpeng You, Maciej Henneberg

**Affiliations:** 1 Biological Anthropology and Comparative Anatomy Unit, School of Medicine, the University of Adelaide, Adelaide, SA, Australia; 2 Institute of Evolutionary Medicine, University of Zurich, Zürich, Switzerland

**Keywords:** cereal availability, ecological study, correlation analyses, wheat, rice, maize

## Abstract

Background: Cereals have been extensively advocated as the beneficial food group in terms of body weight management, but each staple cereal crop may contribute in different ways. Studies of the association between wheat availability and risk of obesity are controversial. This study aimed to test the global and regional association between wheat availability as reported by FAO and obesity prevalence at a population level. FAO does not distinguish between whole grain wheat and refined wheat. Methods: Population-specific data from 170 countries on prevalence of obesity, availabilities of mixed cereals, wheat, rice, maize, meat, sugar, fat, soy and calories and GDP are obtained from the UN agencies. All variables were measured as per capita per day (or per year). Each country is treated as an individual subject. SPSS v. 22 is used to analyse these data for all the 170 countries and official country groupings (regions) using non parametric and parametric correlations, including partial correlation analysis. Results: Pearson's correlation coefficient analysis showed that obesity prevalence is positively associated with wheat availability (*r* = 0.500, *p* < 0.001), but is inversely associated with availabilities of total cereals (*r* = −0.132, *p* = 0.087), rice (*r* = −0.405, *p* < 0.001) and maize (*r* = −0.227, *p* = 0.004). These associations remain in partial correlation model when we keep availabilities of meat, fat, sugar, soy, caloric intake and GDP statistically constant. Overall, positive associations between wheat availability and obesity prevalence remain in different regions. Maize and mixed cereal availabilities do not show independent associations with the obesity prevalence. Conclusions: Our study suggests that wheat availability is an independent predictor of the obesity prevalence both worldwide and with special regard to the regions of Africa, Americas and Asia. Future studies should distinguish between possible influence of whole grain and ultra-processed refined wheat products.

## 1. Background

Obesity is a serious global public health problem that needs to be urgently addressed among all populations [1]–[3]. Obesity increases mortality and morbidity risk from various chronic diseases, such as cardiovascular diseases, diabetes, certain types of cancers and musculoskeletal disorders [4]. Despite progress in knowledge of reasons of obesity, some causes for the obesity epidemic and the disparities between population groups are still unclear.

Obesity is caused by a complex interaction between the environment, genetic predisposition and human behaviour [5]. Diet habits have been implicated in the development of obesity as they may bring environmental factor exposures to people, and this relationship between environmental factor exposure and obesity is complex and not completely understood [2],[6]. Food components such as soy products and sugar in our diets were postulated to contribute to obesity [7] and diabetes [8],[9] respectively in addition to a number of factors, such as physical activities [10],[11], diet composition [6],[12] and genetics [13]. Mixed cereals are important sources of many nutrients including dietary fiber, resistant starch, oligosaccharides, trace elements, vitamins, and other compounds of interest in disease prevention, including phytoestrogens and antioxidants [14]. Dietary guidelines recommend the consumption of mixed cereals to prevent chronic diseases and/or their risk factors. For instance, whole-grain mixed cereals, instead of the individual cereal crop, have been extensively advocated as the major food group for healthy body weight management [15]–[21], and their protective role in reducing the risk of chronic diseases [22] including cancer [23],[24], type 2 diabetes [20],[21] and cardiovascular disease has been shown [20],[21].

Wheat makes up a substantial part of the human diet and is the most important food cereal source for humans [25]. Due to the adoption of Western-style diets that contain significant amount of refined cereal products, wheat demand for human consumption is increasing globally, including countries which are climatically unsuited for wheat production [26]. In the recent years, the association between wheat intake and body weight management has been discussed [21],[26]–[35]. Wheat is provided for human consumption in different forms, principally unrefined or refined. The different forms of wheat products may have different health effects [26],[29],[36]. Whole wheat has been shown to be beneficial for human health [21], [22]. A number of studies suggested that wheat consumption contributes to obesity prevalence in several ways including its use in energy dense and refined products. In this study, we tested the association between the prevalence of obesity (expressed as a percentage of a particular population with a body mass index (BMI) of 30 kg/m^2^ or higher) and wheat availability at the population level on the basis of most recent complete data published by the United Nations (UN) agencies.

## 2. Methods and Materials

### 2.1. Data sources

The country specific data were collected for this ecological study:

1) The WHO Global Health Observatory (GHO) data on the estimates of prevalence of adult obesity (percentage of BMI ≥ 30 kg/m^2^ in country population, 2014).

The GHO is an initiative of the WHO to share data on global health, including statistics by country and information about specific diseases and health measures. The GHO speciﬁcally assembles prevalence data of the biological risk factors, including obesity, for WHO Member States using standardized protocols (http://www.who.int/gho/ncd/methods/en/).

2) The FAOSTAT data on food availability per capita per day in 2011 of mixed cereals (excluding beer), wheat and products, rice (paddy equivalent), maize and products, total meat, sugar and sweeteners, grand total fat, soy products and grand total calories. These data were abbreviated as cereals, wheat, rice, maize, meat, sugar, fat, soy and total calories respectively in this paper.

The FAOSTAT database disseminates statistical data collected and maintained by the FAO. FAOSTAT data are provided as a time-series from 1961 in most domains through the Food Balance Sheet (FBS, http://faostat3.fao.org/home/E). The FBS presents a comprehensive picture of the pattern of a country's food supply during a specified reference period. The FBS shows for each food item i.e. each primary commodity availability for human consumption which corresponds to the sources of supply and their use. The total quantity of foodstuffs produced in a country added to the total quantity imported and adjusted to any change in stocks that may have occurred since the beginning of the reference period gives the supply available during that period. On the utilisation side a distinction is made between the quantities exported, fed to livestock + used for seed, losses during storage and transportation, and food supplies available for human consumption. The per capita supply of each such food item available for human consumption is then obtained by dividing the respective quantity by the related data on the population actually partaking in it [37]. No separate data on consumption of refined and unrefined wheat products are available.

3) The World Bank data on GDP per capita (USD per year, 2011).

The World Bank dataset measures progress on aggregate outcomes for member countries for selected indicators. GDP per capita is gross domestic product divided by midyear population (http://data.worldbank.org/indicator/NY.GDP.PCAP.CD). GDP is the sum of gross value added by all resident producers in the economy plus any product taxes and minus any subsidies not included in the value of the products. It is calculated without making deductions for depreciation of fabricated assets or for depletion and degradation of natural resources.

WHO, FAO and the World Bank are intergovernmental organizations using specialized information relevant to their respective fields. Their professional personnel should have evaluated these data in consideration of their possible use, e.g. for scientific research and decision making, before they were published. Therefore, the data reporting is as free of bias and error as it can be with government statistics. This means that errors are reduced but some inaccuracies related to reporting quality may still be present in the data. Similar data from the same sources were recently used to analyse the relationships between nutrients and obesity [7],[38] and diabetes [8],[9],[39] in a number of publications.

### 2.2. Criteria for data inclusion

The data were selected in consideration of their fulfilment of 1) completeness of data across all analysed variables, 2) the most updated and recent datasets available, 3) major food types: meat, fat, sugar and refined cereals (wheat) that were indicated in the literature to have relationships with obesity. Barley and rye also contain obesity associated gluten like wheat [40], but we did not include them in our study due to their extremely low availabilities in limited areas in the world. Following these conditions, country-level data on obesity prevalence in 2014, cereal availability in 2011 (mixed cereals, wheat, rice, and maize), and potential confounders (meat, sugar, fat, soy, total calories and GDP) in 2011 were matched. We backdated variables and potential confounders to 2011 to reflect exposure with delayed obesity presentation in 2014. The rationale for this decision is that studies have shown that three years is a practical period to develop obesity and metabolic syndrome after exposure to dietary risks (i.e., high intake of wheat today does not lead to immediate obesity) [41]–[43].

In order to contrast the association between wheat availability and obesity prevalence and availability of other cereal crops, we used the availability data of mixed cereals and the other two staple food cereal crops, rice and maize for comparative analysis.

All the aforementioned data were freely downloaded from the UN agencies websites. No ethical approval or written informed consent for participation was required.

### 2.3. Data analysis

We obtained data for 170 countries that had information required for both obesity and wheat availability in a uniform format. Each country was treated individually and all their availability for other variables information was analysed. In this paper the variables and confounders were only referred to with their abbreviations instead of full names followed by their units. For particular analyses, the number of countries included may have differed somewhat because all information on other variables was not uniformly available for all countries due to unavailability from relevant UN agencies. The minimum sample size is 148 for correlation with soy availability. All the data were extracted and saved in Microsoft Excel^®^ for analysis.

Human diet patterns varying in different food components may be affected by the types of food availability in a particular region, socio-economic status and cultural beliefs. In order to demonstrate that association between obesity prevalence and wheat availability is universal regardless of these factors, countries were grouped for correlation analyses. The criteria for grouping countries are UN macro geographical regions, the World Bank income classifications, WHO regions, countries sharing specific characteristics like geography, culture, development role or socio-economic status, like Latin America and the Caribbean (LAC), Organisation for Economic Co-operation and Development (OECD), Asia-Pacific Economic Cooperation (APEC), Southern African Development Community (SADC), the Arab World, Latin America (LA), European Union (EU) and Asia Cooperation Dialogue (ACD). All the country listings are sourced from their official websites for matching except LA which is self-classified based on region primarily speaking romance languages. Countries included in LA are Argentina, Bolivia (Plurinational State of), Brazil, Chile, Colombia, Costa Rica, Cuba, Dominican Republic, Ecuador, El Salvador, Guatemala, Haiti, Honduras, Mexico, Nicaragua, Panama, Paraguay, Peru, Uruguay, and Venezuela (Bolivarian Republic of).

SADC, ACD, LA and EU are included as the sub macro UN continents of Africa, Asia, Americas and Europe respectively to further investigate the correlation within the succeeding macro areas. We could not select any small international organization within Oceania due to very limited number of countries with accessible data. In our analysis, we only included those countries for which we could access the data for the specific groupings.

We calculated the standard deviations of wheat availability and obesity prevalence in United Nations macro continents to explore the variation in Pearson coefficients between wheat availability and obesity prevalence due to the different geographic distributions of country groupings.

Pearson's correlation coefficient and non-parametric correlation coefficient (rho) of Spearman were calculated between all studied variables. Furthermore, partial correlation analysis was conducted keeping some variables statistically constant. All analyses were conducted using SPSS v. 22 (SPSS Inc., Chicago Il USA). In this study, significance was kept at the 0.01 level (2-tailed).

**Table 1.** Sources of country grouping criteria and correlation between obesity prevalence and wheat availability in different country groupings

Country groupings	Pearson's		Nonparametric		Source of country grouping criteria
	r	Sig.	rho	Sig.	
Worldwide (n = 170)	0.500	<0.001	0.555	< 0.001	http://who.int/en & http://faostat3.fao.org
**UN macro geographical regions**					
Africa (n = 47)	0.790	< 0.001	0.767	< 0.001	http://unstats.un.org
Americas (n = 35)	0.518	0.001	0.604	< 0.001	http://unstats.un.org
Asia (n = 42)	0.616	< 0.001	0.639	< 0.001	http://unstats.un.org
Europe (n = 39)	0.222	0.174	0.222	0.173	http://unstats.un.org
Oceania (n = 7)	−0.053	0.911	0.214	0.645	http://unstats.un.org
**Sub-continents within UN macro geographic regions**					
Sub-Africa: SADC (n = 14)	0.633	0.015	0.770	0.001	http://www.sadc.int
Sub-Asia: ACD (n = 26)	0.592	0.001	0.729	< 0.001	http://www.acddialogue.com
Sub-Americas: LA (n = 20)	0.567	0.009	0.502	0.024	Self-classified based on region primarily speaking romance languages
Sub-Europe: EU (n = 28)	0.283	0.144	0.298	0.124	http://europa.eu
**World Bank income classifications**					
Low (n = 32)	0.219	0.228	0.196	0.282	http://data.worldbank.org
Low middle (n = 42)	0.307	0.048	0.544	< 0.001	http://data.worldbank.org
Upper middle (n = 48)	0.257	0.078	0.196	0.181	http://data.worldbank.org
High (n = 48)	0.196	0.181	0.076	0.608	http://data.worldbank.org
**WHO regions**					
AFRO (n = 40)	0.679	< 0.001	0.745	< 0.001	http://www.afro.who.int
AMRO (n = 35)	0.518	0.001	0.604	< 0.001	http://www.paho.org/hq
EMRO (n = 15)	0.285	0.252	−0.010	09.68	*www.emro.who.int*
EURO (n = 50)	−0.002	0.989	0.012	0.933	*www.euro.who.int*
SEARO (n = 10)	0.408	0.241	0.413	0.235	*www.searo.who.int*
WPRO (n = 17)	0.455	0.067	0.613	0.009	*www.wpro.who.int*
**Various economic and cultural country groupings**					
APEC (n = 17)	0.640	0.006	0.689	0.002	http://www.apec.org
Arab World (n = 17)	0.427	0.087	0.140	0.593	http://data.worldbank.org
LAC (n = 32)	0.481	0.013	0.583	< 0.001	http://www.unesco.org
OECD (n = 34)	0.316	0.069	0.176	0.320	http://www.oecd.org

Pearson correlation coefficients and Nonparametric Correlations are reported.

Obesity prevalence is expressed in percentage of defined population with a body mass index (BMI) of 30 kg/m^2^ or higher. Wheat availability is in g/capita/day.

Abbreviations: LAC, Latin America and the Caribbean, OECD, Organisation for Economic Co-operation and Development; APEC, Asia-Pacific Economic Cooperation; SADC, Southern African Development Community; LA, Latin America; EU, European Union; ACD, Asia Cooperation Dialogue.

## 3. Results

In general, obesity prevalence is in significant positive association with wheat availability (*r* = 0.500, *p* < 0.001), but inversely with rice availability (*r* = −0.405, *p* < 0.001) (Table 2). It is also inversely associated with maize availability (*r* = −0.227, *p* = 0.004) and mixed cereals availability (*r* = −0.132, *p* = 0.087) (Table 2).

We subsequently performed nonparametric correlations with the same set of data to test whether the Pearson's correlations between obesity prevalence and all variables differ due to potentially abnormally distributed variables (Table 2).

**Figure 1.**
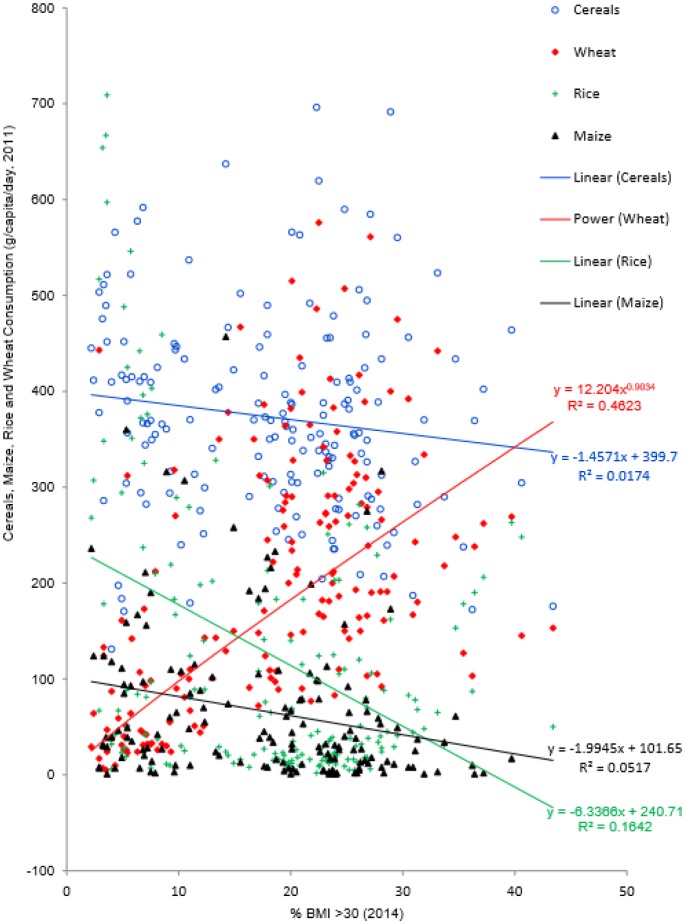
Relationships between obesity prevalence and cereals, maize, rice and wheat availabilities respectively

Figure 1. presents the relationships between obesity prevalence and each cereal food type. Relationships between obesity prevalence and availabilities of total cereals, rice and maize are linear, and wheat availability shows power relationship with obesity prevalence.

When the potential confounders which are the availabilities of meat, sugar, fat and soy, the intake of calories and GDP are controlled for in partial correlation analysis, obesity prevalence is still in positive association with wheat availability (*r* = 0.368, *p* < 0.001) (Table 3). The association between obesity prevalence and rice availability is relatively strong, but negative (*r* = −0.276, *p* = 0.001). No association between obesity prevalence and cereals availability (*r* = 0.065, *p* = 0.436) or maize availability (*r* = −0.004, *p* = 0.963) is observed (Table 3).

The correlation of wheat availability to obesity prevalence in different country groupings is also observed (Table 1). Within the UN macro geographical regions, Africa (*r* = 0.790, *p* < 0.001), Americas (*r* = 0.518, *p* = 0.001) and Asia (*r* = 0.616, *p* < 0.001) have a significant positive association with wheat availability. The association based on Europe region is positive, but not significant. These trends are also observed in Africa sub-grouping (SADC), Americas sub-grouping (LA), Asia sub-grouping (ACD) and Europe sub-grouping (EU) respectively (Table 1).

For the UN macro region of Oceania (Table 1), sample size is small and variation of wheat availability is limited. This renders correlation coefficients uninformative (Table 4).

The association between obesity prevalence and wheat availability exists in all the country groupings categorized by the World Bank based on per capita GDP.

Based on the WHO region classifications, AFRO (*r* = 0.679, *p* < 0.001) and AMRO (*r* = 0.518, *p* = 0.001) have the significant correlation between obesity prevalence and wheat availability. However, there is nearly no association in Europe. The similar correlation can be observed in Africa sub-grouping (SADC), Americas sub-grouping (LA) and Europe sub-grouping (EU) respectively. Wheat availability is also positively correlated to obesity prevalence in EMRO and SEARO. Lack of correlation in Europe is a result of small variation in obesity prevalence and wheat availability (Table 4).

The general trend that obesity prevalence is positively associated with wheat availability can be observed in country groupings regardless of cultural backgrounds, economic levels and geographic locations of the clustered countries. The trends are also present in two functional alliances, OECD and APEC although the former comprises developed countries only and the latter is comprised of both developing and developed countries.

**Table 2.** Correlation coefficients between obesity prevalence and all variables. Pearson r-s are above the diagonal and Spearman rho are below the diagonal.

	BMI≥30	Total Cereals	Wheat	Rice	Maize	Meat	Total Fat	Sugar	Total Calories	GDP	Soy
BMI≥30	1	−0.132	0.500***	−0.405***	−0.227**	0.624***	0.589***	0.660***	0.593***	0.339***	0.252***
Cereals	−0.172*	1	0.406***	0.164*	0.216**	−0.336***	−0.230**	−0.167*	0.147	−0.234**	−0.025
Wheat	0.555***	0.288***	1	−0.465***	−0.347***	0.293***	0.429***	0.380***	0.574***	0.224**	0.063
Rice	−0.252***	0.052	−0.473***	1	−0.145	−0.349***	−0.392***	−0.308***	−0.280***	−0.246***	0.029
Maize	−0.300***	0.116	−0.418***	−0.021	1	−0.313***	−0.381***	−0.260***	−0.302***	−0.291***	−0.038
Meat	0.637***	−0.325***	0.420***	−0.321***	−0.321***	1	0.811***	0.627***	0.666***	0.648***	0.314***
Total Fat	0.631***	−0.210**	0.550***	−0.363***	−0.437***	0.807***	1	0.613***	0.818***	0.731***	0.270***
Sugar	0.664***	−0.182*	0.466***	−0.250***	−0.337***	0.636***	0.641***	1	0.624***	0.480***	0.343***
Total Calories	0.603***	0.095	0.635***	−0.338***	−0.314***	0.679***	0.823***	0.627***	1	0.615***	0.313***
GDP	0.657***	−0.243**	0.513***	−0.287***	−0.388***	0.833***	0.819***	0.704***	0.757***	1	0.188*
Soy	0.339***	−0.052	0.099	0.072	−0.034	0.317***	0.317***	0.358***	0.319***	0.428***	1

Pearson correlation coefficients and non-parametric correlation coefficients (rho) are reported. Number of countries included in the analysis ranges from 148 to170. *P < 0.05; **P < 0.01; ***P < 0.001

BMI ≥ 30 is for obesity prevalence which is expressed in percentage of defined population with a body mass index (BMI) of 30 kg/m2 or higher. Availabilities of wheat, rice, maize, meat, soy, cereals, sugar and total fat are expressed in g/capita/day. GDP is in per capita USD per year. Total calories is in kcal/capita/day.

**Table 3.** Global independent association between obesity prevalence and total cereals and each cereal food availabilities.

Correlation	Cereals	Wheat	Rice	Maize
Partial correlation (r)	−0.065	0.368	−0.276	0.004
Significance	0.436	< 0.001	0.001	0.963
df	145	145	145	145

Partial correlation coefficients are reported. Keeping intake of meats, total fats, sugar, soy, total calories and GDP constant.

Obesity prevalence which is expressed in percentage of defined population with a body mass index (BMI) of 30 kg/m^2^ or higher. Availabilities of wheat, rice, maize, meat, soy, cereals, sugar and total fat are expressed in g/capita/day. GDP is in per capita USD per year. Total calories is in kcal/capita/day.

**Table 4.** Means and standard deviations of obesity prevalence and wheat availability in UN continents.

Continents	Obesity prevalence			Wheat availability	
	Mean	Std. Deviation		Mean	Std. Deviation
Africa (n = 47)	11.75	7.36		126.13	142.21
Americas (n = 35)	24.49	5.26		154.54	65.06
Asia (n = 42)	14.97	11.07		241.24	163.16
Europe (n = 39)	22.14	2.92		278.92	62.19
Oceania (n = 7)	34.47	6.19		166.57	47.15

Obesity prevalence is expressed in percentage of defined population with a body mass index (BMI) of 30 kg/m^2^ or higher. Wheat availability is in g/capita/day.

## 4. Discussion

The worldwide secular trend of increased obesity prevalence likely has multiple etiologies, which may act through multiple mechanisms. By examining the data collected for 170 countries, we have shown that globally obesity prevalence is significantly associated with wheat availability independent of other food components (total fat, soy products, sugar and meat), total calories and GDP. Although results of ecological analysis must be treated cautiously, our results indicated relationship similar to those found in three recent empirical surveys in children [35], young women [28] and general adults [27].

Early in history, barley and rye were much more prominent as dietary grains. However, during agriculture modernization and evolution of our culture, wheat has been recognized as the finest grain [44]. Wheat has a pleasant flavour, an extensive shelf life and unique properties because of gluten-forming proteins [44]. About 95% of the wheat that is grown and consumed globally is bread wheat (*Triticum aestivum*). Bread wheat is a relatively new species, having arisen in southeast Turkey about 9,000 years ago [25],[45]. Extensive wheat breeding by modern agricultural techniques, such as seed selection, hybridization and radiation, has aimed to increase crop yield, improve quality, diversify the strains and develop disease and insect resistance and tolerance to abiotic stresses. Modern agriculture techniques have made hereditary factors of wheat changed. These changes of genetic material may have brought new substances in modern wheat in comparison to those from wheat decades ago [29],[34].

Wheats are subjected to many different processes during their preparation for human consumption. Whole wheat products are rich in fiber, micronutrients and minerals, and have been shown to be beneficial to human health [21],[22]. Refined wheat products have been considered as desired food in the past due to its purity, but refined wheat contains practically only carbohydrates, which is less beneficial nutritionally [46]. Due to their appearance and good taste, food items containing refined wheat may be over consumed. Since we could not obtain separate data on whole wheat and refined wheat consumption, their respective contributions to obesity should be subject of separate study. There are many varieties of wheat gluten proteins which may have structural, metabolic, protective or storage functions [47]. Analyses of proteins expressed by a wheat hybrid compared to its two parent strains have demonstrated that 5% of proteins in general [48] and 14% in gluten proteins [49] were present in either parent. From the evolutionary perspective, new types of crops or food components, such as soy [7], when massively introduced into human diet, may be able to change human nutritional environment with the consequence of contributing to obesity prevalence [12].

Gluten proteins are the major storage components in wheat and may account for up to 80% of the total cereal protein [50],[51]. Anti-nutrients are natural or synthetic compounds that interfere with nutrient absorption. The gluten complex has been considered as one of the anti-nutrients causing inflammation [29],[34],[52] which has been associated with body weight increase in a number of studies in humans [29],[31],[34] and animals [32]. A couple of studies found that gluten consumption was inversely correlated with BMI increase [31],[53], but the two cohorts in the studies had been under clinical treatments due to celiac disease and Crohn's disease respectively.

In this ecological analysis, wheat contributes to obesity. Wheat has greater energy density than another staple food, rice [27]. For instance, eaten in Asia steamed wheat bread doubles the energy from the same amount of steamed rice [54]. It has been well established that energy-dense diets increase risk of obesity because they tend to increase total energy intake [55],[56]. Since we do not have data on whole wheat and refined wheat availability, it can only be suggested that the observed correlation may be a result of refined grain consumption [57].

European countries are culturally and socio-economically relatively homogenous. In Europe, the correlation coefficient between per capita wheat availability and obesity prevalence does not reach the significant level, though it is still positive in our study. This is most likely due to small variances of wheat availability and obesity prevalence in this region (SD of wheat availability = 62.19, SD of obesity prevalence = 2.92, Table 4) that may reduce the co-variance. It may also be that types of wheat products consumed in Europe differ from those consumed in other parts of the world.

Although the association between wheat availability and the obesity prevalence is not significant in the South East Asia (WHO region), it is significant in macro Asia (both UN and WHO definitions) and the Asia sub-ACD. It may be because the obesity prevalence in South East Asia should be assessed with the region specific BMI ≥ 28, instead of the universal BMI ≥ 30 [58],[59]. The universal obesity determining level (BMI ≥ 30) used by the WHO to calculate the obesity prevalence may not be able to determine the actual prevalence of obesity in that area.

In the modern diet, a majority of cereals are refined by the removal of germ, and bran, so that the remaining endosperm is mostly carbohydrate [60]. Refined endosperm may be metabolized to satisfy human daily energy requirement earlier than the other two macro-nutrients which are fat and protein [12],[61]–[63]. Globally, wheat, rice and maize supply around 93% of total daily energy from cereals and 50% of all food calories [63],[64]. A number of other studies also show that mixed cereals availability is not associated with obesity prevalence. In our study, obesity prevalence has been positively associated with wheat availability, but inversely with both rice and maize availability. These positive and negative associations may have been neutralized which may make mixed cereals appear as the healthy food types for body weight control.

In terms of the association between obesity prevalence and rice availability only, our ecological study findings differ from the results of epidemiological studies in Japanese young women [65], in American Hispanic elders [66] and in Korean adult women [67]. There is a similarity among the three studies that the cohorts were on diet patterns which not only mainly consisted of rice, but also contained significant amount of soy products, such as miso soup, tofu or bean *etc.* Soy products contain anti-nutrients which may alter human metabolism and may contribute to obesity [7]. Interestingly, a cohort of Brazilian adults in Sichieri's studies mainly relied on rice and beans being protected against obesity [68]. The underlying reason may be that the diet pattern for that particular cohort was that of low fat and low energy, or that too much rice and high fibre from rice and beans overcompensated the soy's effect on causing obesity.

There are a few limitations in this study. Firstly, lack of separate data of whole wheat and refined wheat availability. Secondly, although we attempted to remove confounding effects of variables such as GDP, caloric, fat availability *etc.* by means of partial correlation analysis, some confounding factors may still influence correlations we found. Some residual curvilinearity may remain even though a relationship between all studied variables appeared to be linear as indicated by similarity of values of Pearson and Spearman nonparametric correlation coefficients. Secondly, there may be some variables not included in our analysis that influence found correlation. For instance, fruits availability correlated, however poorly, with prevalence of overweight in a similar ecological study [6]. Other possible variables to be considered would be vegetables, especially starch-rich varieties. Thirdly, we could only use an international food database that tracks the general market availability of different food types, not the actual human consumption. There are no direct measures of actual human consumption that can account for food wastage and provide precise measures of food consumption internationally. As Siervo *et al.* analysed [38], food disappearance data may not reflect precisely food available to household individual for consumption. Disappearance data may overestimate consumption by some ¼ of total amount. Finally, the data analysed are calculated per capita in each country, so we can only demonstrate a relationship between food group availability and obesity at a country level. For instance, within a country, relationships between characteristics of individuals and their diets might be different due to adoption of specific diet patterns. Cohort studies exploring the association between different cereal species consumption and obesity prevalence would be useful.

## 5. Conclusions

Associations between obesity prevalence and wheat availability at country and regional levels suggest that wheat consumption might contribute to obesity, probably through energy-dense and refined wheat-based foods typical of the Western diet. Ecological studies emphasize associations, not direct causal relationships, so epidemiological studies may be used to explore the association further.
